# Improving phase ii oncology trial using best observed RECIST response as an endpoint by modelling continuous tumour measurements

**DOI:** 10.1186/1745-6215-16-S2-P63

**Published:** 2015-11-16

**Authors:** Chien-Ju Lin, James Wason

**Affiliations:** 1 MRC BSU, Cambridge, UK

## 

In many phase II trials in solid tumours, patients are assessed using endpoints based on the Response Evaluation Criteria in Solid Tumours (RECIST) scale. Typical analyses are based on the proportion of successes among patients, where success is defined by an observed tumour shrinkage above a pre-defined level, and no new lesions appearing. This discards information contained in the extent of the tumour shrinkage. In many trials, patients are followed-up until progression, and their best observed RECIST outcome are recorded, and used as the primary endpoint. An augmented binary approach has been previously proposed for response at a fixed time after treatment (Wason and Seaman, 2013) which uses continuous tumour measurements to improve the efficiency of the analysis of the binary composite outcome. It has been shown to provide considerable extra precision on the estimated success probability, thus gaining power in single-arm and randomised trials. However, it increases computational burden when more than two follow-up times are used, as it requires multi-dimensional integration. In addition, it cannot be directly applied to the best observed outcome. In this presentation, we modify the augmented binary approach and extend it from using fixed response to best observed response. We assess the properties of the modified augmented binary approach by using simulated data. We also apply this approach to real data from a phase II cancer trial using two endpoints, being final observed outcome and the best observed outcome. We show that the modified approach maintains the efficiency benefits and increases computational efficiency.

